# Levine’s Sign Points to Spontaneous Coronary Artery Dissection in a Healthy Young Male

**DOI:** 10.7759/cureus.24893

**Published:** 2022-05-10

**Authors:** Mahsa Mohammadian, Dhaval Shah, Melvin Santana, Sherif Elkattawy, Shruti Jesani

**Affiliations:** 1 Internal Medicine, Rutgers-New Jersey Medical School/Trinitas Regional Medical Center, Elizabeth, USA; 2 Cardiology, St. Joseph's University Hospital, Paterson, USA; 3 Internal Medicine, Rutgers-New Jersey Medical School/ Trinitas Regional Medical Center, Elizabeth, USA

**Keywords:** acute coronary syndrome, energy booster supplements, levine’s sign, stemi, spontaneous coronary artery dissection

## Abstract

Levine’s sign is a universal sign of ischemic chest pain, defined as an individual holding a clenched fist over the chest that has a low sensitivity but is relatively specific for ischemia. Spontaneous coronary artery dissection (SCAD) is a nonatherosclerotic and a very unusual cause of acute myocardial infarction.In literature,* *it has been more common in young women, postpartum, or with fibromuscular dysplasia. Strenuous exercise is a rare cause of SCAD. We describe a case of a healthy 46-year-old Hispanic male who presented to ER after his morning gym session. The initial EKG was unremarkable. However, due to Levine's sign, a repeat EKG was done and showed hyperacute T waves with J-point elevation in the anterior leads. An immediate coronary angiogram revealed a spontaneous coronary artery dissection in the mid-left anterior descending artery (LAD) segment. Given the resolution of the chest pain and thrombolysis in myocardial infarction (TIMI) 3 flow, no intervention was done. The patient was managed medically with an uneventful recovery. In the current times, with the advent of high sensitivity troponin along with other rapid multimodality imaging techniques, the importance of physical signs and symptoms like Levine's sign has diminished. Yet, they still remain a vital part of patient evaluation. Additionally, SCAD is uncommon in males. However, this patient was consuming energy booster powder that may have predisposed him to the SCAD. In our opinion, Levine's sign still has high clinical value in the right context. We also postulate that energy booster supplements may have serious deleterious cardiovascular effects, and large studies are necessary to understand their full effects on the cardiovascular system.

## Introduction

Acute myocardial infarction can happen in the setting of spontaneous coronary artery dissection (SCAD) in rare cases. SCAD is known to be associated with fibromuscular dysplasia, especially in young women and in postpartum state. Strenuous exercise is another rare cause of dissection in angiographically normal coronary arteries. Our patient was a 46-year-old male who presented with chest pain after heavy weightlifting and energy booster powder use. In the literature review, Levine's sign was noticed on presentation, which has a sensitivity of around 38%, specificity of 78%, and a positive predictive value of 55% for ischemia [1]. ST-segment elevation myocardial infarction (STEMI) and SCAD were eventually diagnosed for the patient and managed conservatively.

## Case presentation

**Figure 1:**
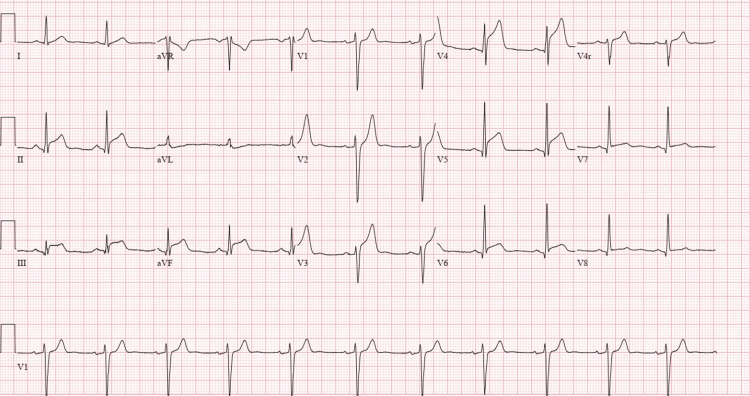
EKG showing ST-elevation in anterior and inferior leads

The patient was a 46-year-old male with a past medical history of essential hypertension who presented to the emergency room with a chief complaint of chest discomfort. The patient stated that he was in the gym and was lifting weights when he started experiencing severe substernal chest discomfort. He had been going to the gym for eight weeks before his presentation. His pain was pressure-like, constant, with an intensity of 6/10, non-radiating, and non-positional. The patient admitted that before exercise, he had used a new energy booster powder for the first time, containing choline, L-citrulline, beta-alanine, betaine anhydrous, agmatine sulfate, L-tyrosine, and caffeine. Levine's sign was noticed by the physician in the emergency room. Initial vital signs were stable with equal normal blood pressure in both arms. First EKG was unremarkable; however, a repeat EKG showed J-point elevation in the anterior precordial leads and inferior leads (Figure 1).

Code STEMI was activated. Initial troponin I was 2.03 (<0.5 ng/ml). A bedside echocardiogram was performed, which showed hypokinesis of the distal anterior, anteroseptal, and apical segment. The patient was loaded with aspirin 325 mg and ticagrelor 180 mg and was taken for cardiac catheterization immediately. Cardiac catheterization showed 100% occlusion of the distal-most segment of the left anterior descending artery (LAD) along with spontaneous artery dissection extending from mid LAD to the distal segment of LAD (Video 1).

**Video 1:** RAO cranial view sharing LAD with dissection in the distal segment, with the distal-most portion of the LAD as the abrupt cutoff Diagonal branches are seen on the side of the true lumen; however, on the side of the false lumen, no septal branches are seen. RAO - right anterior oblique; LAD - left anterior descending artery

It was attempted with a wire to cross the distal LAD, but the wire was not crossing into the distal LAD. At this time, the chest pain was resolved, and thrombolysis in myocardial infarction (TIMI) 3 flow was noticed. Eventually, no percutaneous coronary intervention was performed. Post-procedure, laboratory findings were significant for creatinine 1.90 (0.7-1.2 MG/DL), post catheterization peaked troponin I 55.38 (<0.5 ng/ml), glycated hemoglobin (A1C) 5.1% (4-6.2%), total cholesterol 195 (120-200 MG/DL), low-density lipoprotein (LDL) 164 (<100 MG/DL). Chest X-ray was unremarkable. Medical treatment was continued with aspirin, ticagrelor, transient heparin infusion, metoprolol tartrate, and atorvastatin. Tight blood pressure control was done with nitroglycerin infusion in the first 24 hours. Due to pre-renal acute kidney injury, angiotensin-converting enzyme inhibitors and angiotensin II receptor blockers were avoided initially. Post catheterization echocardiogram revealed a left ventricular ejection fraction of 55% to 60%, apical infarct with severe akinesis at the apex with no defect or clots, mildly increased ventricular wall thickness, and ascending aorta measured 3.6 cm (Video 2).

**Video 2:** Echocardiogram shows apical infarct with severe akinesis at the apex

The patient was clinically stable throughout the hospitalization with uneventful recovery and resolved pre-renal acute kidney injury. A repeat echocardiogram in one month showed no significant changes.

## Discussion

Levine's sign can be seen in 11% of the acute coronary syndrome cases. The sensitivity of the Levine, Palm, Arm and Pointing signs are not exceeding 38% when using troponin levels, functional studies, and coronary angiograms as reference standards. In the literature, Levin's sign specificity is reported between 78% to 86%, but the positive predictive values did not exceed 55%. Currently, with the advent of high sensitivity troponin along with other rapid multimodality imaging techniques, the importance of physical signs and symptoms has diminished [1].

SCAD is a rare non-traumatic, non-iatrogenic separation of the coronary arterial wall resulting in acute myocardium infarction. Proposed mechanisms include intimal tear that causes the separation of coronary wall layers and results in a double-lumen versus vasa vasorum primary disruption with bleeding and intra-medial hemorrhage. Pressure-driven expansion of the false lumen and true lumen compression results in myocardial ischemia [2].

The European Society of Cardiology reported the prevalence of SCAD in 2018 as 0.07-0.2% (roughly 0.1%) of all coronary angiograms performed [3]. Different risk factors are associated with SCAD, including hormonal therapy, postpartum and multiparity, fibromuscular dysplasia (FMD), connective tissue disorders, systemic inflammatory conditions, cocaine abuse, prolonged sneezing, contraceptive pills, and idiopathic dissection.

Pregnancy-related SCAD, more prevalent with multiparty, is a rare but lethal complication of pregnancy, estimated to be happening in 1 in 16,000 pregnancies in the United States. Hormonal changes like estrogen and progesterone surges during pregnancy have been described to be associated with SCAD [4].

Saw et al. studied 50 patients with nonatherosclerotic SCAD over six years. Ninety-eight percent were women with an average age of 51 years. All the patients were screened for FMD. Eighty-six percent of SCAD patients had FMD of ≥1 noncoronary territory. They concluded that nonatherosclerotic SCAD predominantly affects women, and most have concomitant FMD [5].

Intense physical activity can cause ischemic events and may be related to increases coronary shear stress or acute plaque rupture in an atherosclerotic artery. Most cases involve a single vessel, with LAD being the most common one. A survey including details of physical activity and exercise habits before SCAD happened was done on 950 patients from 2011 to 2019 and concluded that 32% of the cases had strength-building exercises before presentation, with more than one cardiovascular risk factor in 70% of the patients [6]. Another observational cohort study of 750 patients with nonatherosclerotic SCAD in North America reported emotional stress as a risk factor in 50.3% and physical stress in 28.9% (about 10% heavy lifting >50 pounds) [7].

Despite multiple studies that demonstrate the association between physical activity and SCAD, the number of SCAD cases that were reported in literature happening after strenuous activity remains low. Kurum et al. reported a case of a young athlete who presented with SCAD after heavy lifting. Spiral dissection of the proximal part of the LAD was found in coronary angiography [8]. El-Sherief et al. reported another case of a 29-year-old male with no past cardiac history who presented with chest pain and was found to have dissection in LAD after the first diagonal/septal branch with extension to the distal LAD that wrapped around the apex. The patient had eight weeks history of heavy lifting prior to presentation [9].

Early diagnosis and management of the SCAD are crucial. Angiographic diagnosis includes the absence of atherosclerotic changes and the presence of radiolucent intimal flap and contrast staining, which is not always present. In uncertain cases, optical coherence tomography (OCT) or intravascular ultrasound (IVUS) can be used.

Management options include conservative management, emergency revascularization with percutaneous coronary intervention (PCI), coronary artery bypass grafting (CABG), or fibrinolytic therapy. Site of dissection, degree of stenosis, single versus multivessel involvement, and hemodynamic state of the patients should be considered. PCI intervention can have a poor prognosis secondary to technical complications and progression of the dissection. Thus, a conservative strategy has been recommended if the patient is hemodynamically stable. In a study done on 182 patients with SCAD by Hassan et al., repeat angiography after 30 days showed complete spontaneous healing in 95% of the dissections [10]. A recent case series in 2021 demonstrated two cases of successful cutting balloon (CB) angioplasty, which is fenestrating the tunica intima and draining the intramural hematoma, an unusual interventional treatment [11].

## Conclusions

Heavy weightlifting is a very rare cause of spontaneous coronary artery dissection, which should be considered in young people presenting with the acute coronary syndrome. We propose that energy powder use with exercise can be another considerable risk factor that needs further investigation and studies. Our case was a demonstration of successful medical and conservative management of a single coronary vessel dissection presenting with STEMI.
